# The influence of community park characteristics on satisfaction in Guangzhou: Moderating and mediating effects analysis

**DOI:** 10.1016/j.heliyon.2024.e31043

**Published:** 2024-05-10

**Authors:** Jia Xin Xiao, Jieying Liao, Bi Zhao, YiLan Long, Xuan Xu, XinYi Liang, Tiansheng Xia

**Affiliations:** School of Art and Design, Guangdong University of Technology, Guangzhou, 510090, China

**Keywords:** Park characteristics, Social self-efficacy, Use intention, Park satisfaction, Older adults, Self-determination theory

## Abstract

Community parks in old urban areas have problems such as outdated facilities and low quality, which inhibit the development of healthy aging. However, limited research has examined the correlation between such park characteristics and park satisfaction among elderly individuals. Additionally, the mechanisms underlying this association remain unclear. In this investigation, a moderated mediation framework grounded in self-determination theory was formulated to probe the interplay between these variables, with a specific focus on the mediating influence of social self-efficacy among the elderly and the moderating effect of use intention. A questionnaire survey (N = 319) was conducted in Shamian Park, Guangzhou, employing structural equation modeling for data analysis. Findings indicate that, even after controlling for demographic factors, park characteristics not only exert a direct influence on satisfaction but also exhibit an indirect impact through the mediation of social self-efficacy, with both pathways being moderated by use intention. This study has implications for how to improve the design of community parks in old urban areas in developing countries to better meet the basic needs of the elderly and promote healthy aging.

## Highlights

• Highlighted basic needs' significance for older adults using Self-Determination Theory
• Built a model examining park characteristics' impact on older adults' park satisfaction.
• Confirmed social self-efficacy's mediating and use intention's moderating roles.
• Offered pertinent suggestions for renovating parks in old urban areas of developing countries.

## 1 Introduction

As the world's second-largest economy, China is currently experiencing a rapid increase in its aging population. Projections indicate that by 2050, China will boast one of the globe's highest percentages of elderly individuals with extended life expectancy [1]. The health and welfare of older adults have been highlighted as a paramount societal concern. Exploring aging through research represents a novel frontier, aiming to harness the capabilities of seniors while addressing their needs and potential contributions. Studies indicate that promoting healthy and active aging is achievable through improved cognitive functioning and effective interventions [2]. Emphasizing the value of well-being is crucial for fostering successful aging [3], aligning with the broader goals of sustainable human development [4].

Recognized as relevant for fostering human health and improving well-being [3], urban green spaces have garnered growing attention in recent years regarding their potential to promote health [5]. Landscape diversity in green spaces helps sustain daily physical activity and has a significant impact on human health [6]. In addition, these spaces offer opportunities to connect with the natural environment [7] and are associated with health benefits through three main pathways: reducing hazards, encouraging physical activity [8], and increasing resilience [9]. Recent research has increasingly focused on enhancing park satisfaction to unlock their health-promoting potential [10]. There is evidence that there is an association between park characteristics (such as location, safety, species richness, and facility maintenance) and users’ park satisfaction (Table 1). However, in these studies, the mechanism of action between the two and the variables that may affect this effect is unclear, and the research subjects are general users. Only a few studies have explored the impact of elderly preferences on satisfaction with community parks in old cities. This is an existing research gap.

**Table 1:** Research on factors affecting park satisfaction.

Author(Date)	Site	Park type	Sample selection	Measured items	Source
van Dinter et al. (2022)	The Netherlands	Urban green parks	Residents	Natural elements, faciliatory elements	[11]
Weijs-Perrée et al. (2019)	The Netherlands	Urban public spaces	Adults	Urban safety, natural elements, air quality, aesthetic quality, smell, accessibility, noise	[12]
Liu & Xiao (2021)	China	Urban parks	Park user	Accessibility, physical attributes, facilities, scenery, management, maintenance	[13]
Saeedi & Dabbagh (2020)	Iran	Urban parks	Park user	Hardscape (architectural elements)	[14]
Arabatzis & Grigoroudis (2010)	Greece	National Park	Visitors	Natural characteristics, Infrastructure, Recreation facilities	[15]
He et al. (2022)	China	Urban green spaces	Residents	Physical perception, aesthetic cognition, psychological cognition, perception of public facilities	[16]
Cernicova-Buca et al. (2023)	Romanian	Green Spaces in Small Urban	Residents	Accessibility, Features, facilities, Uses	[17]
Roberts et al. (2019)	Uk	Park use in a multi-ethnic deprived urban area	Residents	Access, recreational facilities, amenities, natural features, significant natural features, non-natural features, incivilities and usability	[18]
Chu et al. (2021)	China	Urban parks	The older population	Park quality, environmental perception, leisure activity	[19]
Jung et al. (2022)	Emirati	Neighborhood parks	Residents	Park Environments, Accessibility, Park User Behavior	[20]
Maniruzzaman et al. (2020)	Saudi Arabia	Urban parks	Residents	Transport, amenities, environment, safety, accessibility	[21]
Geng et al. (2021)	Canada	National parks	Visitors	Infrastructure, facilities, services, activities	[22]
Halkos et al. (2021)	Greece	Urban parks	Visitors	Relaxation and experience of nature, educational and cultural activities, activities for children	[23]
Quagraine et al. (2022)	Ghana	Urban parks	Park user	Sociability, user and activities, accessibility and linkage, comfort and image	[24]
Hasani et al. (2016)	Iran	Urban recreational parks	Visitors	Sociocultural variables, park functionality	[25]

Old cities, serving as hubs of early commerce and cultural interactions in numerous developing nations, typically boast prime geographical locations [26]. These cities still bear the marks of bygone eras, showcasing their distinctive historical character. Given their longstanding existence and the prolonged residency of many elderly individuals, these communities are facing significant challenges related to aging. Community parks within old cities often feature cramped layouts, haphazard planning, and outdated infrastructure in need of repair. Consequently, these parks suffer from low quality and lackluster social amenities, potentially failing to fulfill the green space requirements of the elderly [27], thus impeding the promotion of healthy aging. In 2020, it was reported that over 14 million families in China required community revitalization efforts [28]. Subsequently, the “Government Work Report” outlined plans to rebuild 53,000 aging urban communities. This urban renewal initiative aims to address issues related to urban aging and foster sustainable urban development. Moreover, it emphasizes the importance of preserving the original community characteristics and the intrinsic connection for the aging population, highlighting the principles of social inclusivity, and encouraging social engagement and active aging among seniors [29]. Therefore, the provision of green spaces for the elderly within old urban areas to enhance their health and well-being should not be approached solely based on the needs of the general users. Given this, this study introduces self-determination theory (SDT) to better discuss the factors that influence the degree of need fulfillment based on older adults to improve satisfaction with old city parks.

Within the realm of psychology, SDT investigates the interplay between needs and environmental stimuli to comprehend the processes and circumstances that foster success in individual, group, and community activities [30]. According to SDT, the need for autonomy, competence and relevance is considered important for all [31] and are key to improving happiness and satisfaction. The need for autonomy refers to the experience of intention, willingness, and choice when participating in activities. For example, the need for autonomy is satisfied if an older person perceives that he is the initiator of park activities and that this activity is consistent with his intention to use it freely. Competence needs refer to the effective experience of handling challenging situations and feeling capable of completing tasks successfully. For example, the need for competence is satisfied when an older adult feels effective in engaging in social interactions at a community park and feels empowered to achieve results. Finally, the need for relatedness includes feelings of connection with others and the desire to have meaningful connections with groups [32]. There is evidence that increased well-being in older adults results from environmental factors that greatly enhance the satisfaction of relatedness needs [33]. For example, if park features help older adults create warm experiences of connection and provide a sense of belonging, older adults' need for relatedness is satisfied.

Thus, guided by the theory of self-determined basic needs, this study aims to integrate park characteristics, social self-efficacy, and use intention via a survey conducted in community parks within older urban regions. The objective is to synthesize the mechanisms through which these factors collectively impact older adults' satisfaction with parks and to identify fundamental considerations for planning and designing the construction of community parks in older urban areas, ultimately facilitating the promotion of healthy aging among older adults.

## 2 Literature review

### 2.1 Park characteristics and park satisfaction

According to the principle of relatedness needs satisfaction, park characteristics can help the elderly in the community create beautiful connections, so park characteristics should be adjusted according to the substantial limitations faced by the elderly, such as vision and hearing loss [34], slow reaction times [35], physical instability and fragility [36], and concerns about personal safety [37]. These restrictions are likely to exert a notable influence on the frequency of seniors' visits to parks and their overall satisfaction levels. Previous research has shown that park characteristics mainly include three dimensions: spatial characteristics, green characteristics, and gray characteristics [38,39]. Specifically, safety and accessibility are the main considerations for the spatial characteristics of the park. Accessibility encompasses four key elements: locality, accessibility, wayfinding, and circulation, all of which directly impact older adults' use of parks [40]. On the other hand, research has shown that the perceived safety of park environments is not only an important factor influencing older adults' visits to parks [41], but also has important implications in terms of promoting aging in place [42]. Green characteristics mainly refer to biodiversity and vegetation richness, and have functions that are important for human well-being [43,44]. Green spaces with species diversity provide an experience of exposure to nature and offer the best potential for recovery for stressed individuals [45]. Research has shown that exposure to natural vegetation has many health benefits, including increased levels of mental health, reduced cardiovascular morbidity, and increased longevity, among others [46], and plays an important role in increasing park satisfaction among older adults. Gray characteristics mainly include the physical environment and supporting facilities of the park. Effective upkeep and administration of public parks, encompassing cleanliness, provision of hygiene facilities, ensuring safe pathways, and adequate lighting, are crucial factors that influence park use [47,48]. Moreover, studies indicate that fostering active aging necessitates age-friendly infrastructures, including accessible walking paths, sufficient seating areas, and well-maintained trails [[49], [50], [51], [52]]. Therefore, this study proposes hypothesis H1: park characteristics positively impact older adults’ park satisfaction.

### 2.2 Mediating role of social self-efficacy

Self-efficacy is an important concept that promotes the social interaction behavior of the elderly and reflects the competence needs of the elderly. Bandura defined self-efficacy as an individual's personal beliefs about his or her ability to succeed or complete a task in a given situation, and stated that it influences behavior [53]. In the social domain, social self-efficacy specifically refers to the ability to take action to achieve the desired levels of social participation for older adults [54]. The relationship between older adults and society plays a pivotal role in their health and longevity [55]. Particularly following retirement, individuals often encounter challenges such as confinement to their homes, reduced independence in daily tasks, social isolation, diminished quality of life, and heightened mortality risks due to changes in their environment and social relationships [56]. Engaging in social activities can mitigate these risks and enhance the well-being of older adults [[57], [58], [59]]. Previous research has shown that low self-efficacy is strongly associated with decreased levels of socialization and loneliness [60]. Community parks, as places where social connections and social networks can be fostered, are the main places for older adults to socialize [61]. This is an important aspect, as whether social needs are met or not is an important factor influencing park satisfaction [40].

On the other hand, several studies have shown that participation in cluster activities and physical exercise has a positive impact on self-efficacy [62,63]. Parks provide facility features and green environments that provide opportunities and assistance for older adults to engage in social activities [64,65]. According to the social cognitive theory [66], self-efficacy directly and indirectly influences behavioral outcomes through outcome expectations, goals, facilitators, and barriers. Park characteristics may act as facilitators or barriers to influence older adults' social behavioral activities by affecting their level of self-efficacy, which ultimately has an impact on park satisfaction. Therefore, this study proposes hypothesis H2: social self-efficacy serves as a mediating factor in the association between park characteristics and park satisfaction.

### 2.3 Moderating effect of use intention

The predictive effect of park characteristics on social self-efficacy and older adults' satisfaction with park use may also be influenced by other factors, such as use intention. Use intention refers to a user's willingness to use a product or service and can also refer to a user's psychological disposition to produce a certain behavior [67]. Previous research has found that the stronger an individual's intention to perform a behavior is, the greater the likelihood of performing that behavior is [68,69]. The fact that use intention affects activity decision-making is a reflection of older adults' need for autonomy fulfillment. Studies have proven the predictive effect of intention on urban green space behavior [70]. The experience of using urban parks depends not only on the presence of various park features [71], but also on individual's perceptions of urban parks [72]. This also implies that older adults with high intention to use may mitigate the negative effects of undesirable park features on the experience of park activities, whereas older adults with low intention to use are more likely to be affected by park features in their experiences and feelings relating to parks.

On the other hand, intention has been found to be a key factor in an individual's participation in activities and is closely related to self-efficacy [73,74]. Intention is expected to moderate the park characteristics–social self-efficacy relationship, as seniors with low intention to use either do not visit the park or do not participate in group activities, and are unable to enjoy the socialization benefits of good park characteristics, i.e., they are unable to increase their social self-efficacy levels. In contrast, for older adults with high intention to use, good park features may be more likely to stimulate their social self-efficacy because a good social atmosphere and cluster activities provide social support for older adults to engage in socialization and also stimulate their self-confidence [62,75]. Thus, this study proposes hypothesis H3: use intention moderates the relationship between park characteristics and both social self-efficacy and park satisfaction.

### 2.4 Research models and hypotheses

**Fig. 1:**
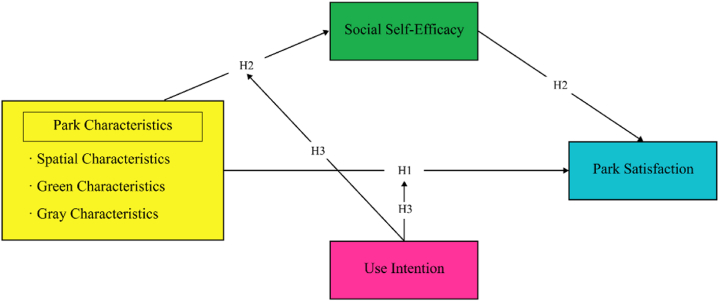
Theoretical framework.

In summary, this study developed a moderated mediation model grounded in self-determination theory to investigate the mechanisms through which use intention and social self-efficacy influence the relationship between park characteristics and older adults' satisfaction with park usage (see Fig. 1). Drawing from existing literature, we formulated three hypotheses: H1: park characteristics positively impact older adults’ park satisfaction; H2: social self-efficacy serves as a mediating factor in the association between park characteristics and park satisfaction; and H3: use intention moderates the relationship between park characteristics and both social self-efficacy and park satisfaction.

## 3 Method

### 3.1 Park selection and participants

This study focuses on community parks as the research subject, employing structural equation modeling to examine the mechanism through which park features (including spatial, green, and gray characteristics) influence the satisfaction of elderly visitors. Additionally, it aims to investigate the potential mediating role of social self-efficacy among the elderly and the moderating influence of park use intention. To enhance the representativeness of the sample and the reliability of the conclusions, according to the Standard for Classification of Urban Green Spaces in the People's Republic of China (CJJ/T85-2017), this study selected Shamian Park in the Liwan District, an old urban community park with the highest degree of aging within Guangzhou City, as the case site. With an area of 21,400 square meters, Shamian Park is located in Shamian, the most exotic area in Guangzhou, which was originally the former embankment garden and queen's garden under the British and French concessions, and has a long and strong cultural history. The park is rich in greenery, with a large open plaza and children's play facilities near the river, making it the first choice for leisure and exercise for the elderly in the nearby community.

Participants were recruited for this cross-sectional study using convenience sampling, and the target population was elderly residents aged 60 years or older who had lived in the community near Shamian Park for more than one year. The questionnaire study was conducted through a cooperative approach with the Guangzhou Society of Gerontology by setting up a booth at the Sha Mian Party Service Station. The questionnaires were distributed in both paper and electronic forms, with the electronic version being distributed and collected by the authoritative Chinese questionnaire website “Questionnaire Star” (https://www.wjx.cn/). Verbal consent was obtained from the participants after confirming eligibility for the study and explaining the purpose of the study, indicating that they agreed to participate in this research study, and they could then begin to fill out the questionnaire. The entire process took about 10 min to complete, and each participant was given a gift (ten dollars’ worth of paper towels) as a reward for participating in the study. For any individual who expressed reading or operational difficulties, the researcher offered to help answer the questions and record the answers. This study received a total of 339 participants to fill in the questionnaire, excluding the 20 invalid questionnaires due to incomplete answers, or not being careful (answering too fast or answers were all the same), a total of 319 valid questionnaires were received, with an effective recovery rate of 94.10 %. The Ethics Committee of Guangdong University of Technology reviewed and validated the ethical procedures of the study (No. GDUTXS2023222).

### 3.2 Research variables

The questionnaire consisted of five sections: demographic characteristics, park characteristics, social self-efficacy, intention to use the park, and park satisfaction (Table 2). The first section asked for background information about the participants, such as age, gender, education level, and frequency of going to the park. The second part used assessment tools from previous studies to measure park characteristics [11,40], which consisted of three dimensions: spatial characteristics (five items), green characteristics (three items), and gray characteristics (six items). Examples include “the park location is close to my home”, “the vegetation in the park is well-maintained”, and “this park has sufficient opportunities to exercise”. In the third section, the Social Self-Efficacy Scale developed by Oki and Tadaka [54] was used to examine the level of older adult's ability to take action to engage socially. The fourth part examines the level of older adults' intention to use the park (with a score of 1 representing not wanting to at all and a score of 5 representing wanting to do very much). Finally, the fifth section assessed older adults' satisfaction with the park via the Life Satisfaction Scale developed by Diener et al. [76]. The questionnaire was assessed using a five-point Likert scale approach, except for the demographic characteristics section (1 for not at all and 5 for completely).

**Table 2:** Variables and items used in the study.

Variable	Scale Item	Source
Demographic Characteristics	Age Gender Educational level How often do you go to the park Whether or not to use mobility aids	–
Use Intention	Level of willingness to use the park	–
Social Self-Efficacy	I can try to go to the park as much as possible to avoid staying withdrawn. I can find a relaxing place in a familiar community park. I can find a little enjoyment in the park. I can use the park's facilities and public services that are useful for my health.	[54]
Spatial Characteristics	The park location is close to my home. The park location is close to public transport. The park was safe. The surrounding community is of a similar social class to mine. The park has a clearly marked entrance.	[77]
Green Characteristics	The variety of vegetation (such as trees, bushes, flowers, etc.) is good. The vegetation in the park is well-maintained. The park is big enough to do the things I want to do.	[11]
Gray Characteristics	This park has enough benches. The quality of the facilities in this park is good. This park has sufficient play facilities for children. This park has sufficient opportunities to exercise. This park has enough walking paths. There is no nuisance from litter in this park.	[11]
Park Satisfaction	In most ways, my experience in the park is close to my ideal. The conditions of the park are excellent. I'm satisfied with my park experience. So far, I have gotten the important experience I want at the park.	[76]

Considering that older participants had some difficulties in conducting the questionnaire, including memory loss, limited reading and comprehension, etc., it was easy for them to lose patience with questions that were difficult to understand, resulting in filling in the answers randomly. In addition, due to the specificity of community parks in old urban areas in China, some questions in the assessment scale of previous studies did not apply to this study, such as “This park provides a convenient parking lot” and “This park is relatively flat and not hilly.” Based on these considerations, the research team carefully adjusted and modified the number and presentation of the questionnaire to limit the number of items to 28, while ensuring a comprehensive assessment of the study variables.

### 3.3 Power analysis

Monte Carlo power analysis was employed to determine the minimum required sample size for the study [78]. With a statistical power set at 0.80 and 10,000 iterations of Monte Carlo simulations with 20,000 samples each, the analysis revealed that at least 146 samples were necessary to detect the effect. This finding underscores the reliability of the sample size chosen for this study.

### 3.4 Data analysis

The questionnaire data were categorized using Excel and invalid data were excluded (e.g., failure to answer all items, high degree of homogeneity of answers across options). Descriptive statistics and correlation analyses were performed for all variables using IBM SPSS Statistics 26.0. Descriptive statistics were used to describe the means and standard deviations of sociodemographic and other variables. The Pearson correlation analysis was used to assess the linear correlation between the two variables. AMOS 24.0 was used to test the proposed framework and assess the path coefficients. Subsequently, SPSS macros PROCESS Models 4 and 8 were tested for mediation and moderation effects. The 5000 bootstrap method was applied, and we utilized 95 % confidence intervals to validate the path effects [79].

### 3.5 Common method bias test

We employed exploratory factor analysis to assess the presence of common method bias in the questionnaire [80]. The findings indicate that the maximum variance explained by the common factor is 36.09 %, which falls below the critical value of 40 %, indicating that there is no common method bias in this study.

### 3.6 Reliability and validity analyses

Table 3 presents Cronbach's alpha values for variables within the park satisfaction model, ranging from 0.863 to 0.891, and composite reliability (CR) values ranging from 0.841 to 0.871. All values surpassed the conventional threshold of 0.7, indicating the reliability of the scale data.

**Table 3:** Reliability and convergent validity.

Construct	Item	Loading	a	CR	AVE
PC	PC1 PC2 PC3	0.639	0.891	0.841	0.553
		0.711			
		0.763			
	PC4 PC5 PC6	0.669			
		0.826			
		0.800			
	PC7 PC8 PC9	0.790			
		0.770			
		0.714			
	PC10 PC11 PC12	0.834			
		0.616			
		0.702			
	PC13 PC14 SSE1	0.763	0.863	0.871	0.631
		0.775			
SSE		0.735			
	SSE2 SSE3 SSE4	0.919			
		0.871			
		0.804			
PS	PS1	0.773	0.881	0.869	0.625
	PS2	0.874			
	PS3	0.905			
	PS4	0.832			

Note: α = Cronbach's alpha; CR = composite reliability; PC= Park Characteristics; SSE=Social Self-Efficacy; PS= Park Satisfaction.

Subsequently, we assessed the questionnaire's validity, encompassing content and construct validity. Since the questionnaire items were derived from established scales, content validity was deemed satisfactory. Structural validity, comprising convergent validity and discriminant validity, was then examined. As indicated in Table 3, the average variance extracted (AVE) was used to assess convergent validity and the AVE for each variable in this study ranged between 0.55 and 0.63 and exceeded 0.5, proving the convergent validity of the model. Additionally, the square root of the AVE values for all variables exceeded their respective correlation coefficients, thus confirming the acceptance of discriminant validity.

In addition, the tolerance and variance inflation factor (VIF) were calculated in this study to assess the multicollinearity of construct-wise and item-wise. The results are shown in Table 4 and all values are within acceptable limits.

**Table 4:** Correlation matrix, discriminant validity, Tolerance and VIF.

Construct	CR	AVE	PC	SSE	PS	Tolerance	VIF
PC	0.841	0.553	**0.744**			0.59	1.68
SSE	0.871	0.631	0.616	**0.795**		0.52	1.91
PS	0.869	0.625	0.673	0.619	**0.791**		

Note: CR = composite reliability; AVE = average variance extracted; VIF=Variance inflation factor; Scores highlighted in bold represent the square root of the average variance extracted for each construct.

## 4 Results

### 4.1 Descriptive statistics and correlation analysis

The demographic results are shown in Table 5. A total of 319 people participated in the experiment, of which 82 (25.7 %) were males and 237 (74.3 %) were females. The largest proportion of older people aged 60–79 years was 88 %, while 11 % were over 80 years old. The most elderly people did not need to use assistive devices to visit the park (95.3 %). In terms of educational background, the majority of older adults had low levels of literacy (84 %), with only a small percentage having earned a bachelor's or master's degree (16 %). In addition, older adults visit community parks more frequently (close to 70 % visit more than 3–4 times a week).

**Table 5:** Demographic information of samples (*n* = 319).

Demographic Dimension		Frequency
Gender	Male	82 (25.7 %)
	Female	237 (74.3 %)
Age	60–69 years	158 (49.5 %)
	70–79 years	123 (38.5 %)
	Above 80 years	38 (11.0 %)
Whether you need auxiliary equipment to travel	Yes	15 (4.7 %)
	No	304 (95.3 %)
	Junior high school	118(37.0 %)
Education background	High school	150(47.0 %)
	Bachelor's degree	49(15.4 %)
	Postgraduate and above	2(0.6 %)
	Once or twice a month	57(17.9 %)
How often do you go to the park	Once or twice a week	39(12.3 %)
	3-4 times a week	69(21.6 %)
	Once every day	154(48.3 %)

The Pearson correlation analysis was used to explore the potential relationship between park characteristics, social self-efficacy, use intention, and park satisfaction, with the descriptive statistical result presented in Table 6. There was a two-by-two significant positive correlation between park characteristics, social self-efficacy, use intention, and park satisfaction.

**Table 6:** Descriptive statistics and correlation analysis (*n* = 319).

	M	SD	1	2	3	4
1. Park Characteristics	4.14	0.74	–			
2. Social Self-Efficacy	4.31	0.96	0.62**	–		
3. Use Intention	4.11	0.96	0.35**	0.48**	–	
4. Park Satisfaction	4.20	0.83	0.67**	0.62**	0.49**	–

Note: *p < 0.05,**p < 0.01. M: Mean. SD: Standard deviation.

### 4.2 Mediation effect test

**Fig. 2:**
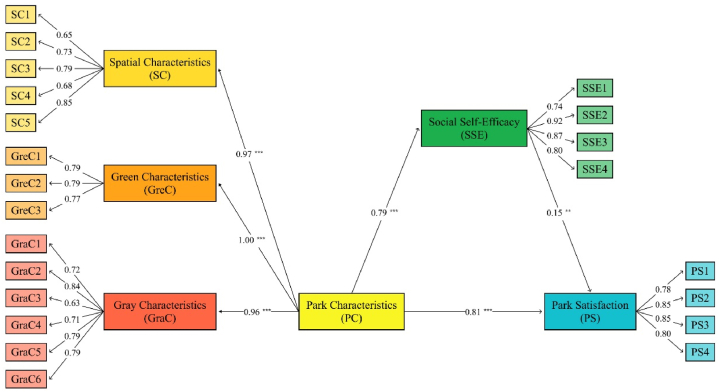
Structural model results. Note: **p < 0.01; ***p < 0.001.

Initially, the model fit was assessed, yielding the following indices: CMIN/DF = 2.98, CMIN = 585.48, DF = 196, CFI = 0.977, TFI = 0.932, and RMSEA = 0.07, indicating an acceptable model fit. The relationships between variables are depicted by the path coefficients, where the magnitude of these coefficients effectively represents the degree of influence between different variables. Based on previous research, in the current study, we included age, gender, education level, and frequency of park visits as control variables in the analysis. The results indicated that the effects of these control variables on the dependent variable were not significant; therefore, further analysis was not conducted. The results of parameter estimation in the park satisfaction model are shown in Fig. 2. The results show that these hypotheses are validated. Specifically, it confirms that park characteristics have a direct effect on park satisfaction (β = 0.81, p < 0.001) and positively affect social self-efficacy (β = 0.79, p < 0.001). Social self-efficacy positively affects park satisfaction (β = 0.15, p = 0.016).

Regression analyses showed that park characteristics significantly and positively predicted both park satisfaction (b = 0.72, p < 0.001) and social self-efficacy (b = 0.77, p < 0.001). Additionally, social self-efficacy significantly and positively predicted park satisfaction (b = 0.30, p < 0.001). The mediation effect analysis demonstrated (Table 7) that social self-efficacy mediated the relationship between park characteristics and park satisfaction, with a mediation effect of 0.20, which accounted for 21.79 % of the total effect of park characteristics on park satisfaction (0.91) (Table 8).

**Table 7:** Mediating effect of social self-efficacy between park characteristics and park satisfaction.

	Social Self-Efficacy			Park Satisfaction		
	b	se	t	b	se	t
constant	1.13	0.18	6.21***	0.12	0.16	0.77
Park Characteristics	0.77	0.04	17.78***	0.72	0.05	14.34***
Social Self-efficacy				0.26	0.05	5.66***
*R*^2^	0.50			0.69		
*F*	316.12			351.59		

Note: ***p < 0.001; SE: standard error.

**Table 8:** Bootstrap 95 % confidence interval of the mediating effect path.

	Effect	Boot SE	Boot LLCI	Boot ULCI	Mediation Effect
Indirect Effect	0.20	0.05	0.11	0.31	21.79 %

Note: Boot SE: bootstrap standard error; Boot LLCI: bootstrap lower limit confidence interval; Boot UULCI: bootstrap upper limit confidence interval.

### 4.3 Moderated mediation test

**Fig. 3:**
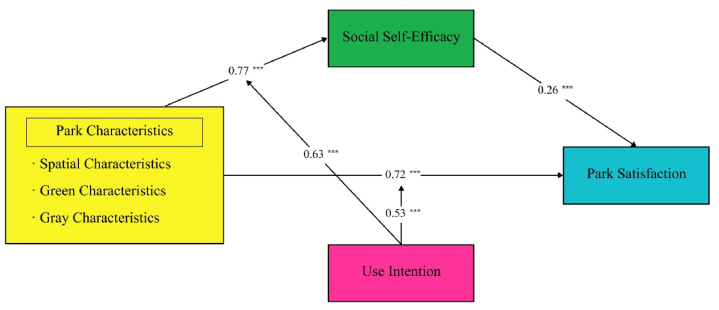
Mediating effects of park characteristics on park satisfaction. Note: **p < 0.01, ***p < 0.001.

As anticipated, we hypothesized that use intention would moderate the relationship between park characteristics and park satisfaction. The findings (refer to Table 9 and Fig. 3) revealed that park characteristics significantly and positively influenced both social self-efficacy (b = 1.05, p < 0.001) and park satisfaction (b = 1.03, p < 0.001). Moreover, social self-efficacy significantly and positively impacted park satisfaction (b = 0.15, p < 0.01). Additionally, use intention moderated the link between park characteristics and park satisfaction (95 % CI [−0.1547, −0.0167], excluding 0), indicating a significant moderating effect.

**Table 9:** Moderation of the mediating effect of use intention on social self-efficacy.

	Social Self-Efficacy			Park Satisfaction		
	b	se	t	b	se	t
constant	−0.84	0.67	−1.26	−1.39	0.55	−2.53^a^
Park Characteristics	1.05	0.17	6.09^c^	1.03	0.15	6.88^c^
Social Self-efficacy				0.15	0.14	3.15^b^
Use Intention	0.63	0.17	3.71^c^	0.53	0.14	3.72^c^
Park Characteristics × Use Intention	−0.10	0.04	−2.46^a^	−0.09	0.04	−2.44^a^
*R*^2^	0.57			0.73		
*F*	136.59			211.95		

Note.

a *p* < 0.05.

b *p* < 0.01.

c *p* < 0.001; *se*: standard error.

**Fig. 4:**
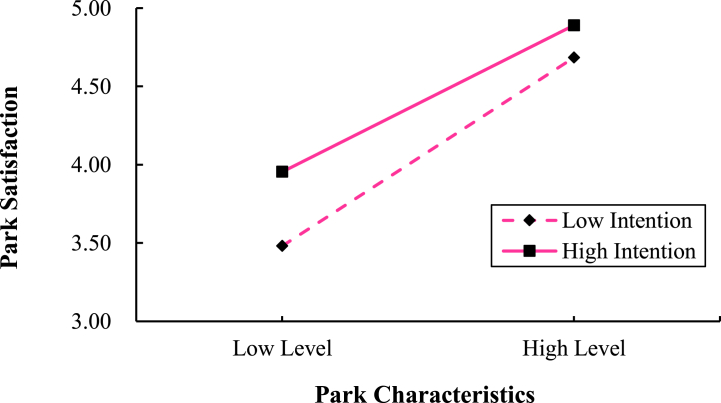
Interaction between park characteristics and use intention affects park satisfaction.

Simple slope tests (Fig. 4) revealed that for high-use intenders, the effect of park characteristics on park satisfaction was significant (b = 0.60, p < 0.001). For low-use intenders, the effect of park characteristics on park satisfaction was also significant (b = 0.77, p < 0.001), but the effect of park characteristics was larger than that of high intentionality, suggesting that higher intention to use parks attenuated the effect of park characteristics on park satisfaction.

**Fig. 5:**
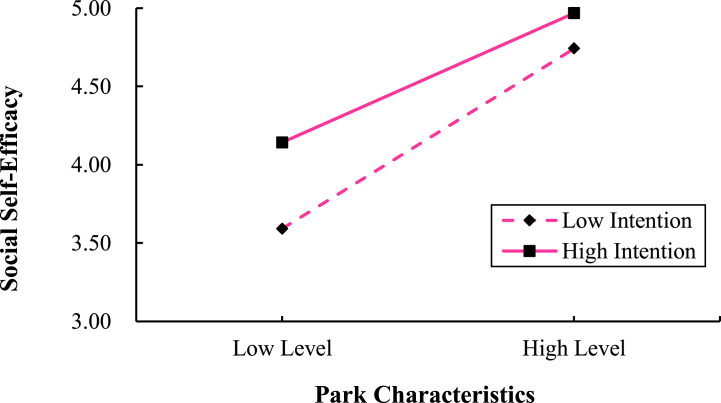
Interaction between park characteristics and use intention affects social self-efficacy.

In addition, simple slope tests (Fig. 5) also indicated that the effect of park characteristics on social self-efficacy was significant (b = 0.53, p < 0.001) for high-use intenders. For low-use intenders, the effect of park characteristics on social self-efficacy was also significant (b = 0.74, p < 0.001), but the effect of park characteristics was larger than that of high intentionality, suggesting that higher intention to use parks attenuated the effect of park characteristics on social self-efficacy.

Conditional analysis of indirect effects further suggested that the moderating role of use intention extended to the indirect influence of park characteristics on park satisfaction through social self-efficacy (95 % CI [−0.1333, −0.0052], excluding 0), as illustrated in Table 10. Specifically, for high-use intenders, there was a small significant indirect relationship between park characteristics and park satisfaction (indirect effect = 0.07, SE = 0.03, 95 % CI = [0.0048, 0.1361]). For low-use intenders, the indirect relationship between park characteristics and park satisfaction (indirect effect = 0.20, SE = 0.04, 95 % CI = [0.0939, 0.2388]) was significantly stronger; therefore, Hypothesis 3 was supported. In other words, social self-efficacy and use intention play a moderating mediating role between park characteristics and park satisfaction, forming a moderated mediation model.

**Table 10:** Bootstrap results of use intention.

Moderator	Boot Indirect Effect	Boot SE	95 % CI	
			Lower	Upper
M-SD	0.1598	0.0373	0.0939	0.2388
M + SD	0.0709	0.0338	0.0048	0.1361

Note: Number of bootstrap samples: 5000. SE: standard error; CI: confidence interval.

## 5 Discussion

Assigning Shamian Park in the Liwan District, Guangzhou City, as a case study of community parks in old urban areas, this study constructed a moderated mediation model between park characteristics, social self-efficacy, use intention, and park satisfaction, and systematically examined the formation mechanism and influencing factors of park satisfaction among older adults. The results show that good park characteristics positively predicted park satisfaction, social self-efficacy mediated park characteristics and park satisfaction, and use intention moderated this mediation path, and the research hypotheses were all supported. An important contribution of this study is that it provides an empirical case for effectively using the self-determination theory framework to explain the importance of basic need satisfaction (autonomy, competence, and relatedness) in improving park satisfaction among older adults while simultaneously providing valuable reference for the renovation of community parks in old urban areas and further enriches the theory and practice of urban ecological service systems in developing countries.

### 5.1 Effect of park characteristics on older adults’ park satisfaction

The present study found that excellent park characteristics positively predicted older adults' park satisfaction levels, which is consistent with previous findings [40,81]. Parks with safe and accessible spatial features, green features with abundant vegetation, and gray features with good amenities were significantly associated with park satisfaction among older adults. As highlighted by multiple studies, residing in age-friendly community settings featuring versatile open spaces conducive to social interaction can foster positive social engagements among older individuals [27]. Such environments cater to their need for relatedness and contribute to heightened life satisfaction [82]. The research underscores the importance of conserving the original community elements and cultural heritage when renovating old urban areas. This approach fosters a greater sense of belonging and identity among residents [83]. According to Wong and Yu [84], older individuals often prefer seated positions during social interactions. Therefore, ensuring ample and comfortable seating can enhance their sense of well-being and self-regulation through interpersonal contact and conversation, thereby affording them more chances to cultivate their social lives and forge deeper social bonds [40]. Schmidt et al. [82] emphasized that diverse levels of social engagement can be facilitated through a range of activities in public open spaces, including dancing, walking, and exercising. Therefore, the renovation of community parks in old urban areas should not only respond to the declining abilities of older people in terms of function and motivation, but also help them create connections by providing age-friendly environments and infrastructures.

### 5.2 Mediating role of social self-efficacy

This study found that older adults' social self-efficacy mediates the relationship between park characteristics and park satisfaction, and that good park characteristics increase older adults' satisfaction in using parks by increasing individuals' levels of social self-efficacy and thus satisfaction. Although previous studies do not have direct evidence of a park characteristics–social self-efficacy–park satisfaction relationship, several studies have shown that park characteristics play an important role in encouraging social interactions and increasing self-efficacy among older adults [63,64], and indirectly influence park satisfaction [40]. The present study, which examined all three variables simultaneously, demonstrated that social self-efficacy mediates the relationship between park characteristics and park satisfaction. The findings support the hypothesis of self-determination theory [85] as well as social cognitive theory [86] that park features provide environmental and amenity support for older adults to engage in social activities, and that social mastery experiences contribute to older adults' mastery of social skills, which may translate into positive evaluations of social self-efficacy, in turn increasing satisfaction with park use. Furthermore, certain scholars have acknowledged the correlation between heightened social interactions in urban green spaces, such as parks, and capacity enhancement, particularly among vulnerable demographics like older adults. Additionally, utilization of urban green spaces has been linked to enhanced self-perceptions and overall well-being [87], increased life satisfaction [88], and the promotion of healthy aging [89].

### 5.3 Moderating effect of use intention

The present study found that use intention not only moderated the relationship between park characteristics and park satisfaction, but also moderated the mediating pathway of park characteristics–social self-efficacy–park satisfaction dynamic.

Specifically, the direct predictive effect of park characteristics on park satisfaction was more pronounced in individuals with high intention to use parks than in those with low intention to use parks. This may be because individuals with high intention to use parks are more intrinsically motivated, i.e., driven by intrinsic factors such as personal interest, curiosity, self-development, and so on, which is another important aspect of self-determination theory [30]. Psychologists have found that when individuals are intrinsically motivated, they exhibit (1) greater interest, enthusiasm, and self-confidence, which lead to improved performance, creativity, and perseverance [31]; and (2) an increased sense of vitality [90] and self-esteem [91], which lead to a general increase in well-being [92]. Further evidence suggests that when older adults are intrinsically motivated to participate in leisure activities, they perceive fewer constraints, display greater engagement, and report higher satisfaction levels during park visits [93]. This implies that intrinsic motivation may be an important factor in improving the level of older adults' use intention to visit parks. Future research will hopefully deepen the understanding of older adults’ motivations to use parks. Emphasizing these elements in park planning and activity design, and improving their use intention by increasing motivation to pursue activities, will lead to a greater degree of autonomy need satisfaction among older adults.

On the other hand, it was found that park characteristics were more likely to adversely affect social self-efficacy and lead to lower park satisfaction in individuals with low-use intention than in individuals with high-use intention. This result suggests that use intention, as a self-regulatory capacity, can play a moderating role in the influence of other variables on an individual's beliefs about his or her ability to perform an activity (social self-efficacy). Previous research has shown that when an individual's basic needs are not met, it can lead to negative controlling behaviors [85]. This means that when older adults feel pressured to do things they do not want to do, they may feel frustrated with their need for autonomy and, in turn, insecure about their abilities and relationships. Competence frustration includes older adults feeling defeated, incompetent, and personally inadequate, while interpersonal frustration can leave older adults feeling disrespected, rejected, and lonely [94]. That is, older adults with low intention to use are more likely to feel insecure about their social competence and are more dependent on the support provided by environmental factors to help increase perceived confidence in performing the behavior.

Thus, older adults with low intention to use are more likely to be influenced by park characterizing conditions, whereas older adults with high intention to use may be less likely to be influenced by changes in park characteristics and tend to make relatively independent judgments.

### 5.4 Research suggestions and limitations

This study reveals the effects of park characteristics on park satisfaction and the mechanisms by which social self-efficacy and use intention play a role in the relationship between the two. Such insights are crucial for enhancing the planning and design of community parks in older urban areas in developing countries, thereby better catering to the needs of older adults, fostering their social well-being, and promoting healthy aging. On the one hand, it underscores the importance of considering the broader influence of community parks on fulfilling the basic psychological needs of older adults. Designers and planners should not only base the provision of facilities and environmental modifications on the physical limitations of older people to compensate for the lack of autonomy and competence needs [36,37]. In addition, it is important to focus on the relevance needs and usage preferences of older people to promote their social connectedness and sense of belonging, thereby promoting social inclusion and cohesion within the community. On the other hand, the findings reveal that use intention has a moderating effect on older adults' perceived competence and experience of use. This requires community organizations or volunteers to provide relevant social support for low-intention individuals to carry out cognitive interventions, such as organizing rich activities to help low-intention older adults cultivate hobbies and interests, and providing psychological counseling to strengthen their self-confidence in perceived behavioral control. Only by fully motivating older adults to participate and increasing their intention to use can we effectively realize the important role that park features can play in improving older adults’ life satisfaction.

In addition, this study has some limitations that could be improved in future research. First, this study conducted the main analysis via a questionnaire survey, but it involves an elderly user group. The use of interview, observation, or focus group research methods can help gain a richer understanding of the range of the fulfillment of their needs [77], and a variety of survey methods should be considered in future studies to synthesize judgments and draw more comprehensive conclusions. In addition, the applicability of this study to community parks in other regions needs to be further explored, considering the differences in geography, community development, and cultural beliefs in different cities.

## Ethical statement

This work has obtained approval from both the Departmental Ethics Committee and the Institutional Review Board of Guangdong University of Technology (No. GDUTXS2023222).

## Funding

This research was funded by grants from the Philosophy and Social Sciences Fund of Guangdong Province (GD24CYS04), the 10.13039/501100001809National Natural Science Foundation of China (52008114), and Research on the development-oriented city of youth in Foshan (2024-QNFZ34).

## Data availability statement

All data are available from the corresponding author upon a reasonable request.

## CRediT authorship contribution statement

**JiaXin Xiao:** Writing – original draft, Funding acquisition, Conceptualization. **Jieying Liao:** Writing – original draft, Investigation, Formal analysis. **Bi Zhao:** Writing – original draft, Funding acquisition, Conceptualization. **YiLan Long:** Investigation. **Xuan Xu:** Investigation. **XinYi Liang:** Investigation. **Tiansheng Xia:** Writing – original draft, Conceptualization.

## Declaration of competing interest

The authors declare that they have no known competing financial interests or personal relationships that could have appeared to influence the work reported in this paper.
